# Original Research Work on Pruritus Ani

**Published:** 1917-12

**Authors:** Dwight H. Murray

**Affiliations:** Syracuse, N. Y.


### ORIGINAL RESEARCH WORK ON PRURITUS ANI

By Dwight H. Murray, M.D., Syracuse, N. Y.

Dr. Murray read his seventh annual report on original research work in Pruritus Ani.

He said that he did not feel it necessary to continue to report the work in as great detail as in past years, because so many men in the profession, over such a wide area, were uniformly reporting a confirmation of his claims for the etiology being streptococcus facalis.

He said that he is working with Parke, Davis & Co. for the perfection of a standard stock vaccine that can be used by anyone without the trouble to make cultures. He has used some of this stock of poylvalent strain from eight successfully treated patients, and the patients on whom it was used seemed to improve. He has used it only one month, therefore full judgment must be suspended until it has had a longer trial.

During the past year he found the patients all getting worse at about the same time and finally found that a laboratory worker had been deceiving him as to the strength and kind of vaccine; and kind of bacteria were used and few, if any, streptococcus facalis. The laboratory worker resigned soon after this and on getting the work in the hands of a proper man all went well again.

Dr. Murray reported that the work of the past year had still more strongly proven the correctness of the bacterial (streptococcus fecalis) infection theory as the etiology. His conclusions of the work are:

First: Conclusions of former years are confirmed and most of them are strengthened by experience of the past year.

Second: The troubles I have had with the laboratory worker, as shown herein, give proof that the benefit received by patients, following the use of streptococcus fecalis vaccine, is not a coincident.

Third: Increasing proof that if rectal pathology is present with sertococcic infection of the anal skin, an operation will not cure the pruritus ani.

Fourth: Increasing proof that if rectal pathology is present without a streptococcus infection of the anal skin, an operation will cure the pruritus ani.

Fifth: Continued proof that they may be complicating infections of the skin, in pruritus ani, by staphylococcus or D. coli.

Sixth: Having published six years of research work, taking into account the report of physicians in this country and abroad who have confirmed his findings as to the skin infection, he feels justified in now claiming that the etioilogy of pruritus ani is a skin infection and that the streptococcus fecalis is the usual bacterium.

